# Predicting involvement of polycomb repressive complex 2 in direct conversion of mouse fibroblasts into induced neural stem cells

**DOI:** 10.1186/s13287-015-0045-x

**Published:** 2015-03-21

**Authors:** Moein Yaqubi, Abdulshakour Mohammadnia, Hossein Fallahi

**Affiliations:** Department of Medical Biotechnology, National Institute of Genetic Engineering and Biotechnology (NIGEB), Tehran, Iran; Department of Molecular Biotechnology, National Institute of Genetic Engineering and Biotechnology (NIGEB), Tehran, Iran; Department of Biology, School of Science, Razi University, Kermanshah, Iran; Medical Biology Research Center, Kermanshah University of Medical Sciences, Kermanshah, Iran

## Abstract

**Introduction:**

Mouse fibroblasts could be directly converted into induced neural stem cells (iNSCs), by introducing a set of known transcription factors (TFs). This process, known as direct reprogramming, is an alternative source of NSCs production for cell therapy applications, hence, more common sources for such cells including embryonic stem cell (ESCs) and induced pluripotent stem cell (iPSCs) are also in use. Despite their importance, the exact role of different TFs involved in the conversion of fibroblasts into iNSCs and the interactions between these factors has not been studied.

**Methods:**

Here, we have used available microarray data to construct a gene regulatory network to understand the dynamic of regulatory interactions during this conversion. We have implemented other types of data such as information regarding TFs binding sites and valid protein-protein interactions to improve the network reliability. The network contained 1857 differentially expressed (DE) genes, linked by11054 interactions. The most important TFs identified based on topology analysis of the network. Furthermore, in selecting such TFs, we have also considered their role in the regulation of nervous system development.

**Results:**

Based on these analyses, we found that *Ezh2*, *Jarid2*, *Mtf2*, *Nanog*, *Pou5f1*, *Sall4*, *Smarca4*, *Sox2*, *Suz12*, and *Tcf3* are the main regulators of direct conversion of mouse fibroblasts into iNSCs. Because, members of the polycomb repressive complex 2 (PRC2) were present in the most effective TFs’ list, we have concluded that this complex is one of the major factors in this conversion. Additionally, gene expression profiling of iNSCs, obtained from a different data sets, showed that *Sox2* and *Ezh2* are two main regulators of the direct reprogramming process.

**Conclusions:**

Our results provide an insight into molecular events that occur during direct reprogramming of fibroblasts into iNSCs. This information could be useful in simplifying the production of iNSCs, by reducing the number of required factors, for use in regenerative medicine.

**Electronic supplementary material:**

The online version of this article (doi:10.1186/s13287-015-0045-x) contains supplementary material, which is available to authorized users.

## Introduction

Embryonic stem cells (ESCs) and induced pluripotent stem cells (iPSCs) are the two main sources of induced neural stem cells (iNSCs) generation. Recently direct reprogramming has been introduced as an alternative approach to produce NSCs for use in regenerative medicine. In this approach, over-expression of a set of pre-defined transcription factors (TFs) reprograms fully differentiated fibroblasts into neural stem cells (NSCs) and neurons. Production of iPSCs from somatic cells and their subsequent differentiation into NSCs is a rather slow process (one or two months for each step). Direct reprogramming, on the other hand, is a much faster approach to generate NSCs. This process involves only one step, while generating NSCs via iPSCs requires first reprogramming and then differentiation. In addition, cells produced via ESCs and iPSCs show different degrees of genetic instability and harbor cancer development risks [1].

Recently, several groups have produced NSCs and neurons from terminally differentiated cells, including fibroblasts [2-9], sertoli cells [10], and astrocytes [11]. Interestingly, neurons were also produced from fibroblasts [12-19] by introducing certain types of TFs or a combination of TFs and miRNA [18,19]. Two different approaches have been successfully used for direct reprogramming. One uses four well-known TFs: OCT4, KLF4, SOX2, and MYC similar to Yamanaka’s method, in production of iPSCs. In this procedure, additional factors are required for generation of NSCs and neurons, which should be applied before iPSCs clonal formation [20]. In the second approach, direct conversion of fibroblasts into NSCs was achieved by introducing a list of alternative TFs. Systematic elimination of TFs resulted in identification of a minimum set of TFs that are essential for successful direct conversion in this method [20].

Thus, previous findings highlight the role of TFs in differentiation, fate specification, and direct reprogramming. Fortunately, computational methods are able to predict the most important TFs involved in such cellular processes [21,22]. To this aim, a gene regulatory network has been constructed for mouse ESCs using expression and TFs binding data [23]. However, the dynamics of the gene regulatory network during direct conversion of fibroblasts into iNSCs has not been studied.

Here, we have constructed a gene regulatory network for conversion of mouse fibroblasts into iNSCs and investigated the role of differentially expressed TFs in this process. We have used publicly available data obtained from wet-lab experiments, including microarray expression profiles, information regarding TFs binding sites and valid protein-protein interactions to construct our gene regulatory network. Statistical analysis of this network unveiled a central role for several TFs. Finally, we have extracted and introduced the most important TFs that are involved in regulating the conversion of fibroblasts into iNSCs.

## Methods

### Microarray availability and analysis

Microarray data for direct conversion of adult mouse fibroblasts into iNSCs was obtained from the Gene Expression Omnibus (GEO) using GSE31598 accession number [2]. Raw data were normalized using the robust multi-array averaging (RMA) algorithm in the FlexArray [24]. Differentially expressed (DE) genes were detected by comparing the gene expression profiles of mouse fibroblasts and iNSCs, using a fold change algorithm implemented in the FlexArray software [25]. A very restrictive fold change = 3 was set as the threshold for detection of DE genes, in order to eliminate background noises. Annotation of the probe sets was achieved using annotation file number HG-U133_Plus_2.na33.annot, which was obtained from [26].

The original data were enriched by incorporating gene expression profiling obtained from independent but related studies conducted by Thier *et al*. [3], Han *et al*. [4], and Ring *et al*. [5] (Table 1). Mouse annotation files, MouseWG-6_V2_0_R3_11278593_A, MouseRef-8_V2_0_R3_11278551_A, and MoGene-1_0-st-v1.na34.mm10.probeset were used for annotating the data of Thier *et al*., Han *et al*., and Ring *et al*., respectively.

**Table 1:** **Microarray data sets that were used in this study and their experimental design**

**Experiment**	**Comparison**	**Accession number**	**Description**
Matusi *et al*. (2012) [2]	Adult fibroblasts-derived neurosphere (iNSC1) versus adult skin fibroblasts	GSE31598	INSC1 sample is very similar to EB-derived secondary neurosphere. This comparison was used as primary data for our analysis
Thier *et al*. (2011) [3]	Three iNSC colons versus mouse embryonic fibroblasts (MEFs)	GSE36484	iNSC2, iNSC3, and iNSC5 colons compared with MEF.
Han *et al*. (2012) [4]	5 F-iNSCs versus mouse fibroblasts	GSE30500	Best result for direct conversion achieved when combinations of five TFs were used.
Ring *et al*. (2102) [5]	Two clonal lines of iNSCs versus mouse embryonic fibroblasts (MEFs)	GSE37859	Direct reprogramming of mouse fibroblast into iNSCs using Sox2 transcription factor.

In addition to direct conversion of fibroblasts to NSCs, we compared expression profiles of converting astrocytes and neurons to NSCs to find the role of identified TFs in cell fate specification. To this aim, microarray data sets obtained from Deng *et al*., [27], Kim *et al*., [28] and Cahoy *et al*. [29] were used (Additional file 1: Table S1).

### Functional classification of DE genes

Affected cellular pathways and processes were identified using DAVID (Databases for Annotation, Visualization and Integrated Discovery) [30,31] by browsing the DE genes obtained from comparison of iNSCs and fibroblasts in this database. Altered functional clusters were ranked according to the enrichment scores returned by DAVID. Enrichment scores higher than 1.3 (*P*-value <0.05) were considered as highly significant.

### Construction of TFs regulatory network

The binding sites of TFs on the genomic DNA were extracted from the chromatin immunoprecipitation (ChIP) Enrichment Analysis (ChEA) database, the primary depository information for ChIP-chip, ChIP-seq, ChIP-PET, and DamID experimental data. The manually curated section of this database accommodates 458,471 TF-target interactions for 200 TFs. The DE gene list, resulting from microarray analysis, was used as the query list for the ChEA website [32]. Resulting TFs with a *P*-value of 0.05 or lower and a two-fold change in expression were then considered as differentially expressed TFs (DE-TFs). Next, data regarding protein-protein interactions for the DE-TFs list were obtained from the BioGRID database [33]. The most significant protein-protein interactions were identified using the expression profiles of the interacting counterparts. In construction of the TFs regulatory network and TFs protein-protein interaction network only valid and significant protein-protein interactions have been used. Finally, data obtained for TFs-regulatory sequences, protein-protein interactions, and expression profiles were combined to construct a holistic TFs regulatory network in Cytoscape [34].

### Identification of affected biological pathways during direct programming

To identify the most significant biological processes affected in the transition of fibroblasts to iNSCs, one could look at the number of DE genes in each process. Here, we have used two plugins of Cytoscape, namely ClueGO and CluePedia, to find enriched biological pathways in the network. A two-sided hyper-geometric approach (Enrichment *versus* Depletion) was used as a statistical test to calculate the enrichment score and *P*-value using the Bonferroni step down method, implanted in the ClueGO and CluePedia plugins [35,36].

### Evaluation of network by motif analysis

In any network, motifs are a small group of interacting nodes that occur in higher number in the regulatory networks compared to that of random networks. To find three-node motifs in the directed (where direction of interaction is determined) and colored (where upregulated and downregulated genes are specified) regulatory network, we used fast network motif detection (FANMOD) software. The significance of any identified motif was evaluated by its *z*-score and *P*-value. This tool calculates *z*-score through computation of the differences between motif occurrence in the regulatory network and that of random networks (the program by default produces 1,000 random networks using the nodes) [37,38].

### Identification of the central genes and modules in the regulatory network

Central genes in the constructed gene regulatory network were identified using CentiScaPe, a plugin of the Cytoscape software [39]. A set of network related parameters, known as centrality parameters, including degree of connectivity, eccentricity, closeness, betweenness, stress, and centroid were measured. The most important genes were ranked based on the results obtained from these parameters and, consequently, the most central TFs regulating such genes were identified.

The TFs interaction network was also subjected to this analysis using the MCODE plug-in of the Cytoscape [40]. From such analysis the most central protein complexes that are involved in direct conversion of the fibroblasts into the iNSCs were extracted.

Different parts of the networks show different degrees of activity. This is true for the expression network, where some parts of the network show higher expression (called active modules) compared to other parts. To identify such active modules in the gene regulatory network, we have used JActiveModules [41]. JActiveModules uses *P*-values of the differentially expressed genes to find the most active group of genes in the regulatory network [41], therefore, we have loaded these values for the DE genes alongside expression data on the network.

## Results

### Differentially expressed genes and affected pathways during direct conversion of fibroblasts to iNSCs

By comparing gene expression profiles of fibroblasts and iNSCs [2], we have identified several DE genes, which are involved or affected through reprogramming of fibroblasts. Collectively, 2,167 DE genes were identified, of which 1,020 were upregulated and 1,147 were downregulated (Additional file 2: Table S2). Next, in order to identify which cellular processes are affected, functional clustering of these DE genes was conducted. The results indicate that signal transduction is the most affected process, where it contained the maximum number of affected genes. Expectedly, genes related to the nervous system were also among the most affected pathways, where 29 DE genes were involved in the development of the nervous system.

### Identification of TFs that are involved in the regulation of DE genes

Using ChEA, TFs with a role in direct reprogramming of fibroblasts into iNSCs, were identified for the DE genes obtained as described in the previous section. Altogether, 46 TFs were detected that regulate 1,854 of the 2,167 DE genes. We found that 37 TFs were upregulated whereas 9 TFs were downregulated. The highest upregulation was observed for *Pou5f1*, *Zic3* and *Myb*, while *Cebpb*, *Klf2* and *Pparg* were the most downregulated TFs. Finally, we combined all regulatory TFs interactions with those of protein-protein interactions and expression data to construct the regulatory network (with 1,857 nodes and 11,054 edges) (Additional file 3: Table S3).

**Figure 1:**
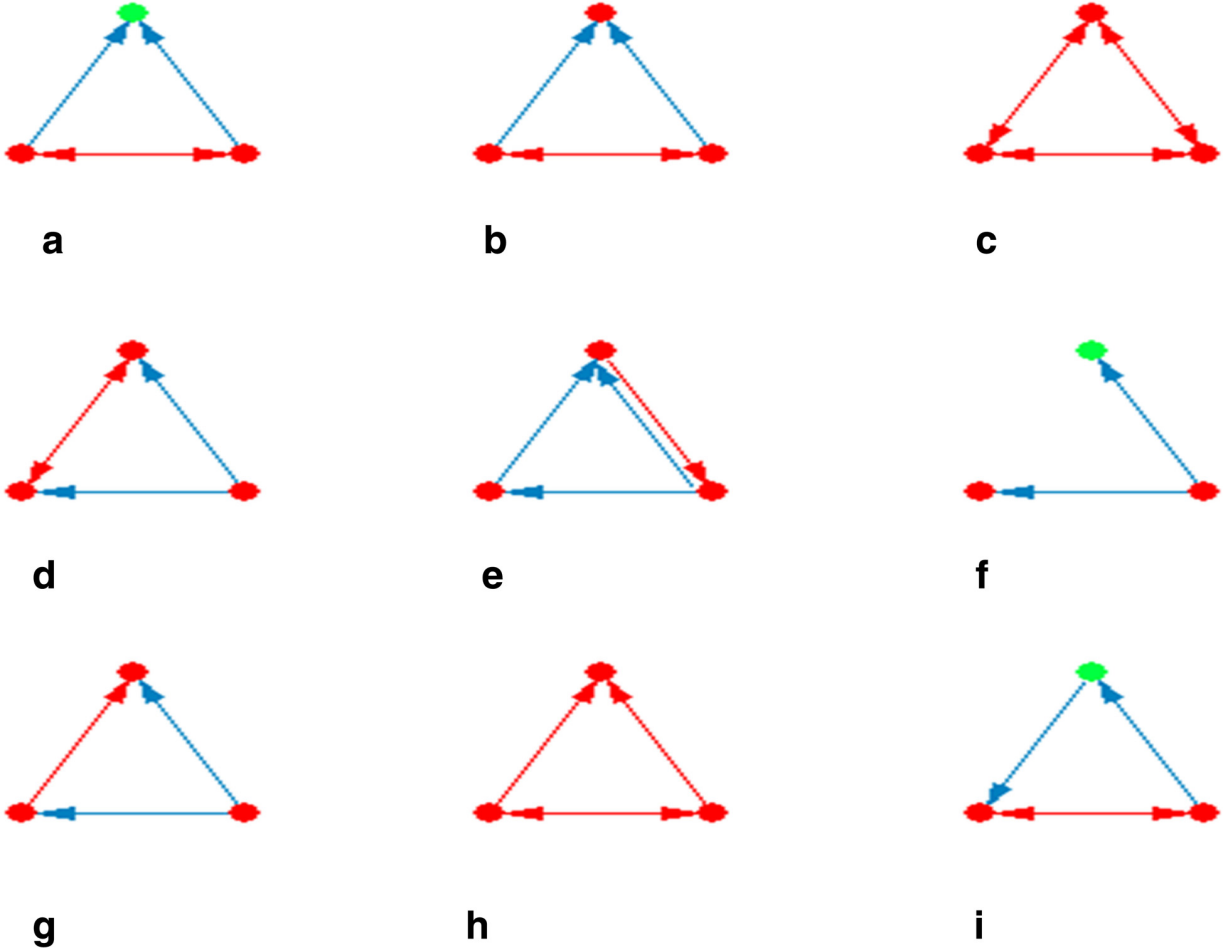
**Enriched three-node motifs in the main regulatory network.** Motifs with *z*-score above two and a *P*-value of zero are presented. Parts **a** to **i** show motifs with the highest frequency in order. A red node indicates upregulation, whereas a green node shows downregulation. Blue and red edges show regulatory and protein-protein interactions, respectively. The arrows show the direction of regulation.

**Figure 2:**
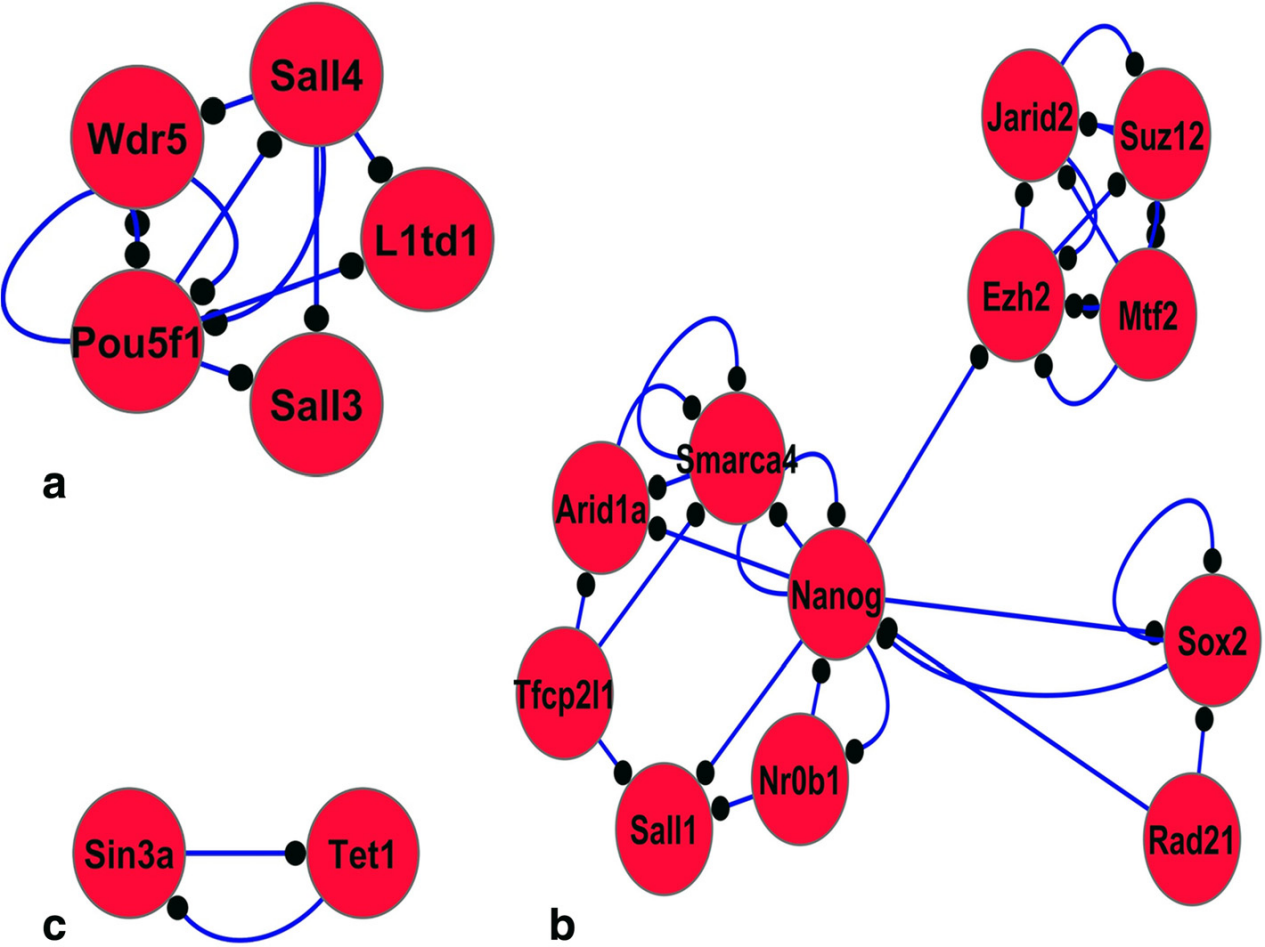
**Identified protein complexes in TFs protein-protein interaction network.** Valid protein-protein interactions used to construct TFs protein-protein interaction network. **a)** to **c)** indicate protein complexes with a score more than 2. Red nodes show upregulation. TFs, transcription factors.

**Figure 3:**
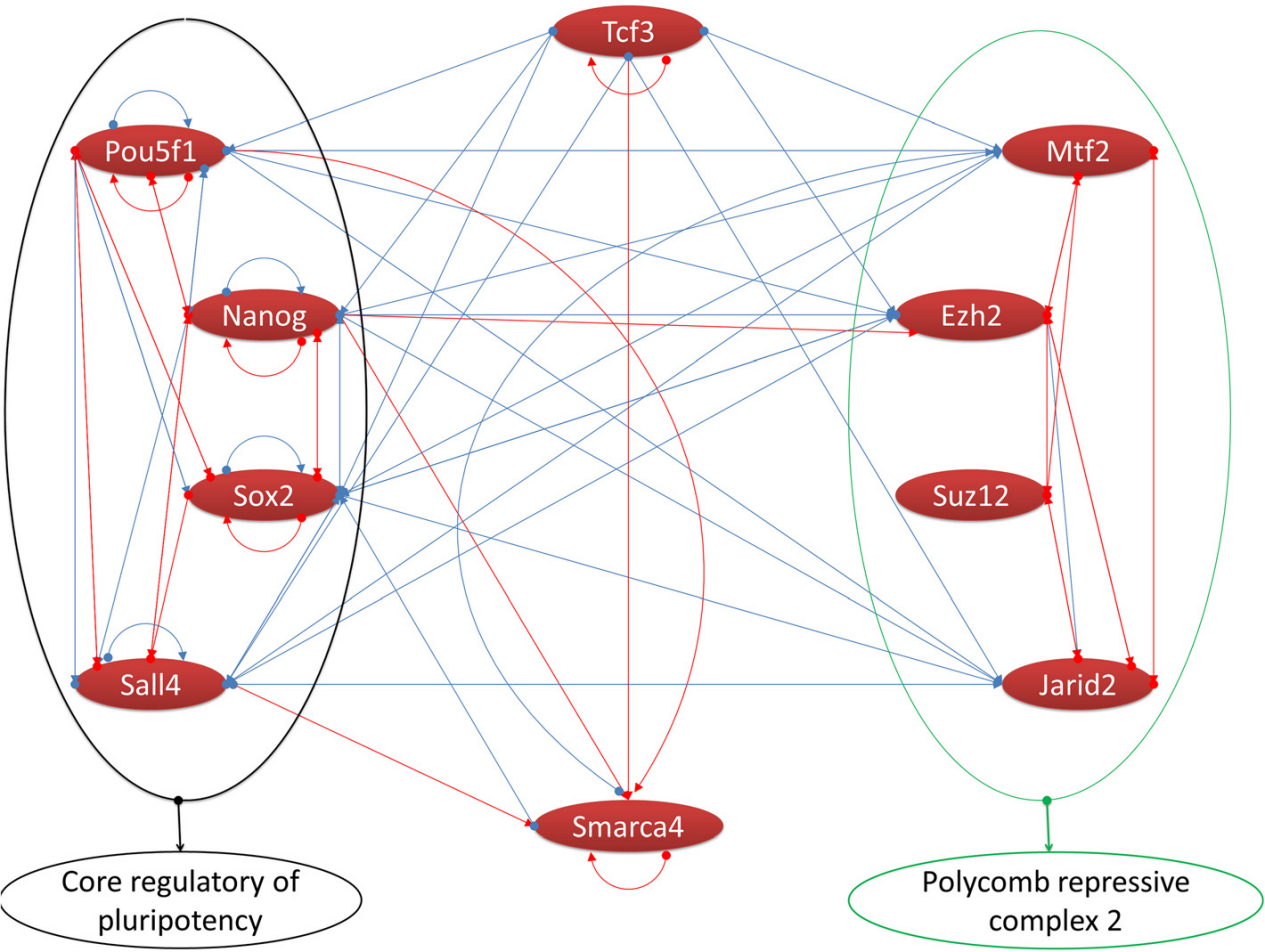
**Regulatory interactions and protein-protein interactions between ten top regulators of direct reprogramming.** These sub-networks accommodate 10 nodes with 70 edges. Red nodes show upregulation. Blue edges show regulatory interactions whereas red edges are protein-protein interactions. PRC2 complex and core regulatory pluripotency members locate in these sub-networks. PRC2, polycomb repressive complex 2.

Motifs are small sub-networks in the core regulatory network that play a role as building blocks of the regulatory network. Generally, they are processing specific information and are involved in special biological tasks. We have identified such important motifs in the TFs regulatory network using the FANMOD tool, where we found 26 motifs (Figure 1). Different interacting patterns were observed between TFs and their targets. The most recurring patterns in our regulatory network were two interacting TFs that co-regulate a third gene (Figure 1a and b). Protein clique (three proteins that interact reciprocally) (Figure 1c) showed high frequencies, for example protein interactions between *Ezh2*, *Suz12*, and *Mtf2* in the formation of the PRC2 (Figures 2b and 3). Co-regulated interacting proteins (Figure 1d) were the next discovered pattern based on their *z*-score. In this motif, one TF regulates two genes, where their protein products interact with each other.

### Analysis of network-related parameters to identify important regulatory components

**Figure 4:**
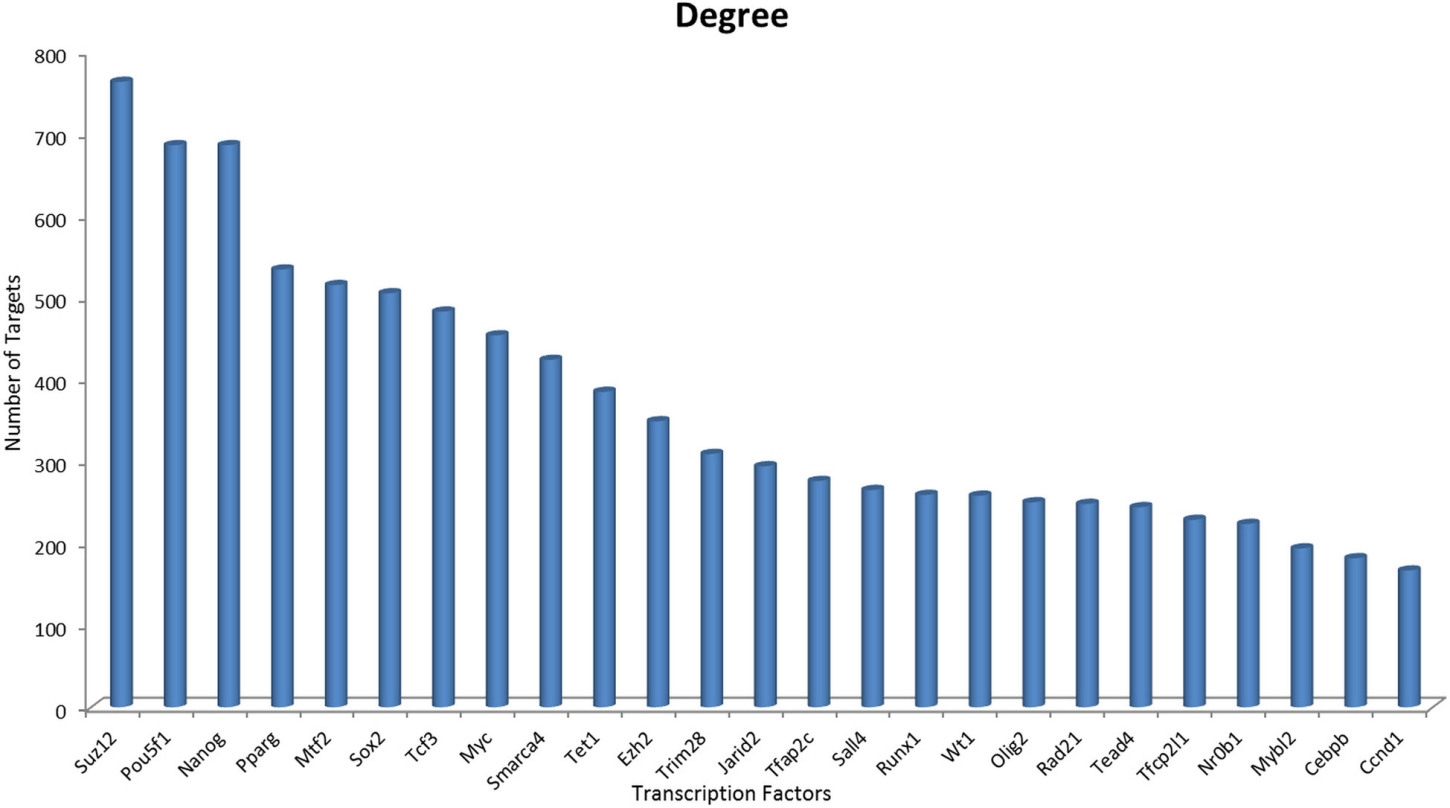
**Connectivity analysis of the gene regulatory network.** Numbers of interactions of the top 25 hub regulators are represented.

To find central TFs, we integrated different sources of data, namely protein-DNA interactions, protein-protein interactions and expression data to build a multi-component gene regulatory network. Based on the connectivity analysis of the gene regulatory network, 15 central TFs *Nanog*, *Pou5f1*, *Pparg*, *Mtf2*, *Sox2*, *Myc*, *Suz12*, *Tcf3*, *Smarca4*, *Ezh2*, *Jarid2*, *Tet1*, *Sall4*, *Tcfap2c*, and *Trim28* were found to be central elements in direct reprogramming of adult fibroblasts into iNSCs (Figure 4). Results of analysis of six network-related centrality parameters are illustrated in Additional file 4: Table S4.

Experimentally validated protein-protein interactions obtained for 43 of these 46 TFs, were used to construct a protein-protein interaction network. This network consisted of 122 proteins and 216 interactions. MCODE was used to investigate if these TFs are involved in regulation of any important protein complexes. We obtained the highest scores for a group of TFs including WDR5, POU5F1, SALL4, SALL3, and L1TD1; all of them showed upregulation during conversion of fibroblasts to iNSCs. The second most important module include NR0B1, SALL1, SMARCA4, ARIDA1, NANOG, RAD21, JARID2, MTF2, SUZ12, EZH2, SOX2, and TFCP2L1; notably these proteins were also upregulated. Except SALL1 and ARIDA1, the rest are among the 46 TFs identified during direct conversion of the mouse fibroblasts into iNSCs. The third motif had only two TFs, TET1 and SIN3A, with just two interactions. Collectively, 14 TFs out of 46 TFs were identified as being present in the protein complexes during direct reprogramming of the mouse fibroblasts to iNSCs (Figure 2).

To find the role of TFs in an active sub-network based on the expression pattern of DE genes, we used JActiveModules. TFs, such as *Cebpb*, *Stat4*, *Suz12*, *Rad21*, *Tcf3*, *Gata3*, *Tfcp2l1*, *Wt1*, *Olig2*, *Ezh2*, *Jarid2*, *Nanog*, *Sall4*, *Sox2*, *Nr0b1*, *Myb*, *Zic3*, and *Pou5f1*, were found to be involved in the conversion of the mouse fibroblasts into the iNSCs (Table 2). Based on ontology analysis of three top modules, we also found the presence of the genes related to the neural tube formation process, including *Adm*, *Celsr1*, *Cthrc1*, *Sall4*, *Sox11*, *Zic2*, and *Zic5*. Interestingly, the main regulators of these seven DE genes were *Ezh2*, *Suz12*, and *Nanog*.

**Table 2:** **The most important active modules and their participant regulators in the conversion of mouse fibroblasts into iNSCs**

**Comparisons**	**Active modules**	
	**Nodes**	**Transcription factors**
iNSC versus fibroblast	83	Nr0b1, Suz12, Rad21, Pou5f1, Zic3.
	90	Stat4, Nr0b1, Suz12, Rad21, Jarid2, Sall4, Pou5f1, Myb, Zic3, Gata3.
	182	Nr0b1, Ezh2, Nanog, Rad21, Myb, Zic3, Stat4, Suz12, Sall4, Pou5f1, Sox2.
	213	Nanog, Rad21, Zic3, Suz12, Wt1, Sall4, Cebpb, Tcf3, Sox2, Nr0b1, Ezh2, Myb, Pou5f1, Olig2, Tfcp2l1.
	5	Sall4, Pou5f1, Nanog, Sox2.

iNSCs, induced neural stem cells.

Taken together, based on our statistical analysis implanted in the tools we have used, a number of TFs, including *Pou5f1*, *Sall4*, *Nanog*, *Jarid2*, *Suz12*, *Ezh2*, *Sox2*, *Rad21*, *Mtf2*, *Tfcp2l1*, *Nr0b1*, *Smarca4*, *Tet1*, *Olig2*, and *Tcf3* were found to be present in at least two out of three networks analyzed in the current study.

### The role of TFs in the regulation of nervous system development

Based on the gene regulatory network ontology analysis, we identified 236 DE genes that were involved in the nervous system development process. The most important regulators of this list were *Suz12*, *Mtf2*, *Pou5f1*, *Nanog*, *Ezh2*, *Tcf3*, *Sox2*, *Jarid2*, *Smarca4*, and *Myc*, which were scored based on the number of targets they regulate.

Analysis of the gene regulatory network showed that at least ten signaling pathways are involved in the conversion of the fibroblasts into the iNSCs. The mitogen-activated protein kinase (MAPK) cascade, transmembrane receptor protein tyrosine kinase signaling pathway, G-protein coupled receptor signaling, Wnt receptor signaling pathway that required for basic developmental processes, Ras protein signal transduction, ERK1 and ERK2 cascade, Rho protein signal transduction, transforming growth factor beta receptor signaling pathway, cytokine-mediated signaling pathway, and BMP signaling pathway were all among those pathways that were significantly affected by conversion of the mouse fibroblasts into the iNSCs (Additional file 5: Table S5). Further network analysis of the signaling pathways revealed that *Mtf2*, *Tcf3*, *Suz12*, *Nanog*, *Pou5f1*, *Sox2*, *Pparg*, *Smarca4*, *Ezh2*, *Jarid2*, *Tet1*, *Trim28*, *Sall4*, and *Myc* were the main regulators of the majority of these ten signaling pathways.

By narrowing down the number of the most important TFs, we have successfully identified the key regulators in the network. For example, *Mtf2* was found to regulate 27% of all regulated genes in the constructed gene regulatory network, while this TF regulates 71% of the DE genes in the BMP signaling pathway during direct conversion of the mouse fibroblasts into iNSCs. On the other hand, *Suz12* is involved in the regulation of at least 50% of the DE genes of the Wnt receptor signaling pathway, G-protein coupled receptor signaling pathway, MAPK cascade, transmembrane receptor protein tyrosine kinase signaling pathway and transforming growth factor beta receptor signaling pathway (Table 3 and Additional file 6: Table S6).

**Table 3:** **Predicting collaboration of TFs in regulation of the most affected signaling pathways**

**G.symbol**	**MAPK signaling pathway**	**Transmembrane receptor protein tyrosine kinase signaling pathway**	**G-protein coupled receptor signaling pathway**	**Wnt receptor signaling pathway**	**Ras protein signal transduction**	**ERK1-ERK2 cascade**	**Rho protein signal transduction**	**Transforming growth factor beta receptor signaling pathway**	**Cytokine-mediated signaling pathway**	**BMP signaling pathway**
Suz12	0.56^a^	0.54	0.60	0.63	0.43	0.47	0.46	0.51	0.37	0.67
Nanog	0.51	0.42	0.32	0.44	0.48	0.5	0.53	0.48	0.41	0.60
Mtf2	0.41	0.46	0.43	0.55	0.28	0.33	0.33	0.41	0.34	0.71
Pou5f1	0.44	0.37	0.29	0.5	0.33	0.44	0.33	0.34	0.48	0.60
Tcf3	0.47	0.33	0.31	0.42	0.35	0.47	0.36	0.41	0.27	0.71
Sox2	0.34	0.31	0.25	0.42	0.38	0.33	0.4	0.27	0.27	0.53
Smarca4	0.38	0.30	0.26	0.32	0.25	0.41	0.3	0.37	0.34	0.42
Ezh2	0.26	0.31	0.28	0.38	0.33	0.22	0.33	0.31	0.13	0.67
Pparg	0.34	0.33	0.28	0.28	0.35	0.33	0.3	0.34	0.24	0.42
Jarid2	0.26	0.27	0.26	0.34	0.17	0.22	0.2	0.31	0.20	0.57
Trim28	0.27	0.24	0.21	0.38	0.17	0.22	0.16	0.37	0.24	0.5
Tet1	0.26	0.24	0.25	0.32	0.20	0.25	0.2	0.34	0.27	0.28
Sall4	0.30	0.31	0.10	0.25	0.12	0.41	0.1	0.20	0.17	0.39
Myc	0.23	0.19	0.18	0.23	0.10	0.22	0.4	0.17	0.27	0.25
Tfap2c	0.22	0.22	0.17	0.13	0.12	0.33	0.13	0.10	0.34	0.28
Olig2	0.22	0.06	0.15	0.25	0.12	0.16	0.16	0.31	0.24	0.28
Wt1	0.16	0.21	0.17	0.28	0.10	0.13	0.1	0.27	0.10	0.321
Rad21	0.16	0.18	0.14	0.21	0.12	0.22	0.13	0.24	0.24	0.17
Nr0b1	0.22	0.22	0.06	0.19	0.10	0.27	0.13	0.10	0.10	0.32
Phc1	0.16	0.19	0.18	0.23	0.07	0.13	0.1	0.13	0.10	0.32

^a^Fraction of differentially expressed genes in each signaling pathways that regulated by given TF. TFs, transcription factors.

By comparing the list of TFs that were involved in the regulation of nervous system development and signaling pathways, we further shortened the list of the most important regulators. It was found that TFs such as *Suz12*, *Mtf2*, *Pou5f1*, *Nanog*, *Ezh2*, *Tcf3*, *Sox2*, *Jarid2*, *Smarca4*, *Myc*, *Trim28*, *Pparg*, *Sall4*, and *Tet1* appear to regulate nervous system development as well as signaling pathways during direct reprogramming of the mouse fibroblasts into iNSCs.

Finally, by combining the results of network analysis with those obtained from biological processes (presented in the previous sections) ten TFs, *Pou5f1*, *Sall4*, *Nanog*, *Jarid2*, *Suz12*, *Ezh2*, *Sox2*, *Mtf2*, *Smarca4*, and *Tcf3*, were found to be the most important TFs during direct programming of fibroblasts to iNSCs (Figure 3).

### Validation of the list of predicted TFs by comparative analysis

Using our pipeline, we have identified 46 DE TFs that are involved in the direct conversion of mouse fibroblasts into iNSCs. Further statistical analyses and regulatory effects on biological processes revealed that ten TFs were the main regulators of direct reprogramming.

To further validate these findings, we used additional cell lines provided by the Matusi *et al*. [2] study. Independent data sets provided by incorporating Ring *et al*. [5], Han *et al*. [4] and Thier *et al*. [3] data into the main set were analyzed (for details please refer to the Methods section).

In the current study, we used epidermal growth factor (EGF)-dependent iNSCs, which were generated from adult mouse fibroblasts, as central data for our analyses [2]. This cell is the best option for treating spinal cord injury (SCI) because a high degree of similarity exists between Embryoid body (EB)-derived secondary neurospheres and neurons, in comparison with other cell lines [2]. For more confirmation of our results, the expression data of three other cell lines from the same study were also investigated [2]. Following the same protocol, 1,911 DE genes were identified. TF binding site analysis using ChEA revealed that 23 of the top 25 TFs are also expressed in the same pattern and are involved in the regulation of direct conversion in these three cell lines as EGF-dependent iNSCs. For example, four members of polycomb repressive complex 2, *Ezh2*, *Suz12*, *Mtf2*, and *Jarid2* are grouped as master regulators in four cell lines based on the number of regulatory interactions and also show the same pattern of expression. The same result also can be deduced for core regulators of pluripotency, *Sox2*, *Pou5f1*, *Nanog*, and *Sall4*, in both regulation of DE genes and expression pattern.

Using the Ring *et al*. data set [5], two clonal lines of iNSCs were compared with wild type mouse embryonic fibroblasts (MEFs). Analysis of expression profiles revealed 1,398 DE genes with a *P-*value less than 0.05. Consequently, we have identified 35 DE-TFs to be involved in the regulation of most of these DE genes. Interestingly, comparison of the top TFs from the Matusi *et al*. data with those obtained from the Ring *et al*. study revealed TFs *Ezh2*, *Mtf2*, *Suz12*, *Tcf3*, *Pou5f1*, *Sox2*, and *Smarca4* as the key regulators in both studies based on the connectivity analysis. In addition to these regulators, *Sall4*, one of the top ten TFs in analysis of the Matusi *et al*. data, ranked 12th in the Ring *et al*. data analysis. The expression pattern of these TFs is similar in both studies, in which all of them were upregulated during direct conversion of mouse fibroblasts into iNSCs.

Using the Han *et al*. data set [4], we identified a set of 1,158 DE genes during conversion of the mouse fibroblasts after application of five TFs. Using their DE genes lists and ChEA analysis we identified 38 TFs expressed differentially. By comparing our top ten TFs list with these 38 DE-TFs we found that *Ezh2*, *Jarid2*, *Sox2*, and *Nanog* were present in both analyses.

Similar approaches were taken for Thier *et al*. [3] expression data, which were obtained from comparative study of stably expanded NSCs and MEFs. We found 1,572 DE genes among their data. Using this set of genes we have identified 34 DE-TFs. By comparing the list of 34 DE-TFs with the top ten TFs from our analysis, three DE-TFs, *Ezh2*, *Sox2* and *Tcf3*, showed similar roles in the regulation of DE genes and expression patterns. Interestingly, a comparison of the three analyses showed that two TFs including *Ezh2*, the core catalytic component of PRC2 and *Sox2*, the pluripotency regulator, are the main regulators of direct reprogramming of fibroblasts into iNSCs.

### Overlapping of TFs across different studies

In summary, we analyzed expression profiles of iNSCs and fibroblasts from four independent studies, which used different protocols in the generation of iNSCs from fibroblasts. We compared the list of TFs involved in these four studies to identify common TFs across all of these protocols. Collectively, seven DE-TFs, *Cebpb*, *Ezh2*, *Rad21*, *Rcor2*, *Runx1*, *Sox2*, and *Tead4*, were identified as common TFs across analysis of expression profiles of these different experiments. Interestingly, six of these seven regulators, *Cebpb*, *Ezh2*, *Rad21*, *Rcor2*, *Runx1*, and *Sox2*, showed the same pattern in their expression, but *Tead4* showed a different pattern in Matusi *et al*. and Ring et al., in comparison with Han *et al*. and Thier *et al*. DE-TFs *Ezh2*, *Rad21*, *Rcor2*, and *Sox2* were upregulatd during direct conversion of mouse fibroblasts into iNSCs, but *Cebpb* and *Runx1* were downregulated. In conclusion, comparing the top TFs across all analyses led to the identification of *Ezh2* and *Sox2* as the master regulators of direct conversion of mouse fibroblasts into iNSCs in all experimental procedures.

### *Ezh2* role in cell fate specification of NSCs

We have identified the TFs *Ezh2* and *Sox2* as master regulators of direct reprogramming of mouse fibroblasts to iNSCs. The role of *Sox2* in the direct conversion of mouse fibroblasts into iNSCs was clarified by Ring *et al*., which showed the conversion of fibroblasts to iNSCs using only *Sox2* overexpression [5]. In addition, several studies have used *Sox2* along with other factors to obtain iNSCs from mouse fibroblasts [2-4,6-8]. With regard to *Ezh2*, we have no reports which use this factor to convert fibroblasts to iNSCs. For more confirmation of the role of *Ezh2* in generation and differentiation, we have compared the expression profile of NSCs with neurons and astrocytes.

Wild type samples of Neural progenitor cells (NPCs) and neurons were obtained from the Deng *et al*. study [27]. Microarray data were normalized and genes with a *P*-value <0.05 have been identified as DE genes. Collectively, 1,400 genes were identified to be DE during comparison of the expression profile of NPCs with neurons. Results of the analysis revealed downregulation of *Ezh2* during generation of neurons from NPCs. In addition to expression analysis, TFs binding site analysis revealed a list of TFs and their role in regulation of DE genes. Connectivity analysis showed *Ezh2* as one of the ten top regulators of the generation of neurons from iNSCs. These analyses show *Ezh2* upregulation in NSCs and its importance in cell fate specification of NSCs. These results indicate that *Ezh2*, besides being a master regulator in conversion to extremely different cell types, also plays role in conversion of NSCs to neurons, which is more similar to NSCs.

In addition to comparison of the expression profiles of NSCs with neurons, we have compared microarray data of NSCs with astrocytes which are more similar to NSCs than fibroblasts. The microarray data for this comparison was obtained from the Cahoy *et al*. [29] and Kim *et al*. [28] studies. Normalization and identification of DE genes led to identification of 1,318 DE genes with a *P*-value <0.05. Expectedly, similar to previous results obtained for comparison of NSCs with neurons, *Ezh2* was upregulated in NSCs and downregulated during differentiation toward astrocytes. Furthermore, our analysis on the number of TF binding sites during differentiation of NSCs to astrocytes identified *Ezh2* as one of the ten top regulators of astrocytes generation from NSCs.

In summary, the comparisons show good consistency with the role of *Ezh2* in cell fate specification of NSCs and confirm its importance in the direct conversion of fibroblasts into iNSCs. The downregulation of *Ezh2* during differentiation of NSCs toward astrocytes and neurons was previously confirmed [42]. In addition, analysis of the overexpression of *Ezh2* in astrocytes showed that this factor led to the reprogramming of astrocytes to NSC-like cells.

## Discussion

We have constructed a gene regulatory network for genes and TFs in which their expressions were altered during direct conversion of the mouse fibroblasts into the iNSCs. Potential regulators of the constructed network were identified and ranked based on statistical analyses and their involvement in the biological processes. We have shown that ten TFs, *Pou5f1*, *Sall4*, *Nanog*, *Jarid2*, *Suz12*, *Ezh2*, *Sox2*, *Mtf2*, *Smarca4* and *Tcf3,* are the most influential regulators in this conversion. These TFs are mainly involved in the regulation of developmental processes and pluripotency characteristics. A possible role of the predicted TFs during this conversion is discussed in the following paragraphs.

Enhancer of zeste homolog 2 (Ezh2) is a methylteransferase component of the polycomb repressive complex (PRC2) [43]. PRC2 regulates self-renewal of stem cells, while its enzymatic activity is mediated by Ezh2. Over-expression of Ezh2 has been observed in NSCs and up regulation of this gene can promote differentiation of NSCs to oligodendrocytes [43]. In another study by the same group, the role of Ezh2 in proliferation and conservation of the NSCs properties was established. It has been confirmed that Ezh2 is a determinant factor in the fate of NSCs and its upregulation causes differentiation towards oligodendrocytes, while its down regulation causes neural and astrocytes differentiation [42]. In addition, it was shown that over-expression of Ezh2 in astrocytes leads to reprogramming of these cells into NSC-like cells [44]. Matusi *et al.*, produced iNSCs with the ability to differentiate into glia cells and neurons even in early passages [2]. We have constructed our integrated regulatory network based on microarray data obtained from the Matusi *et al*. study. Based on these observations and our constructed network, we can hypothesize that Ezh2 dedicates gliogenic properties to iNSCs. Therefore, we propose that the expression of this factor in fibroblasts might be useful in the production of iNSCs with a high ability to differentiate into glia cells.

PRC2 contains three components: Ezh2, Suz12, and Eed polycomb proteins. Suz12 and PRC2 directly bind to the promoter regions of many genes that participate in neural differentiation [45]. On the other hand, it has been shown that Suz12 plays a crucial role in ESCs differentiation, but not in ESCs proliferation [46]. Investigation of the role of Suz12^+/−^ and Suz12^−/−^ ESCs upon differentiation to neurons showed that Suz12^+/−^ ESCs successfully differentiate into neurons while no neurons formed during differentiation of Suz12^−/−^ ESCs [46]. Interestingly, in accordance with previous results, we have identified Suz12 as the master regulator of the nervous system development processes during direct conversion of mouse fibroblasts into iNSCs. As Suz12 and Ezh2 are major components of the PRC2 complex, these findings may highlight the role of the PRC2 complex during such conversion.

The association of Mtf2/Pcl2 with the PRC2 complex has been identified by Wlaker *et al*. in mouse ESCs [47]. They used microarray analysis data obtained from Mtf2 knockdown mice to identify affected genes. Their functional clustering of DE genes showed that the upregulated gene list contained ESC associated genes, while downregulated genes were mostly involved in developmental processes, such nervous system development. They also showed that Mtf2 knockdown had a similar effect as those observed for the Suz12 and Ezh2 null ESCs, where higher similarity was observed between Mtf2 knockdown and Suz12 null ESCs [47]. On the other hand, it has been documented that in the absence of Mtf2, differentiation of ESCs has been retarded and the cells retained their pluripotency properties [48]. Our study showed the existence of an association between the function of Mtf2 and that of Ezh2 and Suz12 members of PRC2 during direct reprogramming of the mouse fibroblasts into the iNSCs (Figure 2b). These results show a high consistency with previous studies in the identification of Mtf2 as a regulator of nervous system development. Therefore, we introduce Mtf2 as one of the key regulators of nervous system development, second to the Suz12 protein.

In addition to Mtf2, the Jarid2/Jomunji complex has been identified as another component of PRC2 in ESCs. While the proliferation properties of the stem cells were not affected in Jarid2^−/−^ ESCs, during the differentiation stage a master regulator of pluripotency, Pou5f1, was found to be in excess and consequently differentiation was retarded [49]. Additionally, it has been reported that Jarid2 is essential for precise regulation of gene expression during differentiation, where interruption of this gene leads to similar results as Suz12 knockout cells [50]. It has been shown that guiding PRC2 to its targets depends on the presence of Jarid2 and this process would be disrupted in theJarid2 knockout ESCs. Accordingly, we have identified Jarid2 in the DE-TF list and verified its interactions with Ezh2, Suz12, and Mtf2 in the protein-protein interaction network. Therefore, this TF should be considered as one of the top regulators of nervous system development and signaling pathways.

Sox2 plays a role in sustaining NSC properties as well as their differentiation [51,52]. This TF, in combination with other TFs, was used to convert fibroblasts directly into NSCs [2-8]. Interestingly, Sox2 individually, without the interaction of other TFs, is able to convert the fibroblasts into the NSCs, where it expresses NSC marker genes and has the potency to direct fibroblasts differentiation into neurons, oligodendrocytes, and astrocytes [5]. From the constructed gene regulatory network, we found that Sox2 is one of the main regulators during direct conversion of the mouse fibroblasts into the NSCs. Indeed, this TF, similar to Ezh2, was identified as one of the principle regulators.

Sox2, Pou5f1, and Nanog serve as core regulators in human and mouse ESCs and together they co-regulate the expression of many genes. These TFs regulate pluripotency characteristics of ESCs through upregulation of genes involved in this process. In addition, these factors maintain the pluripotency properties by downregulation of genes that initiate differentiation into three germ layers. Each of these regulators exhibits self-regulations as well as cross-regulations in ESCs [53,54]. It has been shown that sall4 with Pou5f1, Sox2, and Nanog construct a self-controlling network that is crucial for maintenance of ESCs pluripotency and their subsequent differentiation [55]. Sall4 is reported to regulate expression of Pou5f1 and Sox2 through binding to their promoters [55]. It has been shown that Sall proteins, including SALL1, SALL2, and SALL4, are essential for neurolation [56]. Interestingly, Pou5f1 alone or in combination with small molecules is able to convert fibroblasts into iNSCs. For example, Mitchell *et al.*, showed that Pou5f1 alone is sufficient to convert human adult fibroblasts into NPCs, following expression of NPC markers and the ability to differentiate to astrocytes, oligodendrocytes, and neurons [9,57]. Consistent with our results, this factor has been identified as one of the co-regulators of the Pou5f1, Sox2, and Nanog genes based on gene regulatory network analysis of mouse ESCs [23].

In addition to these eight genes, we have also identified Smarca4/Brg1 and Tcf3/Tcf7l1 as minor regulators of direct reprogramming of the fibroblasts into the iNSCs. Interestingly, Smarca4 has been introduced as a chromatin remolding factor, essential for NSCs fate decision between neurogenesis and gliogenesis [58]. Smarca4 gene expression directs NSCs to differentiate into glia cells, while its repression results in neuronal differentiation [58]. Accordingly, we hypothesize that over-expression of both Ezh2 and Smarca4 might be useful in the production of iNSCs with potency to differentiate into glia cells. Tcf3 is a TF that makes a connection between the Wnt signaling pathway and the core regulatory network of pluripotency in ESCs upon differentiation. ESCs knockdown for Tcf3 showed an upregulation of core pluripotency genes (Pou5f1, Sox2, and Nanog) followed by its inability to differentiate [59]. It is possible that Tcf3 determines the balance between stemness and differentiation [59]. Previously, it was found that Nanog expression increases in the absence of Tcf3 [60]. Tcf3 plays a role in neural tube proliferation and maintaining of progenitor cells identity. Spinal progenitor cells deficient for Tcf3 showed disruption of progenitor properties despite maintenance of proliferations [61]. Interestingly, this factor used in combination with other TFs to convert the fibroblasts into the NSCs. Replacing of Pax6 and Olig2 with Tcf3 resulted in highest conversion efficiency [4].

In summary, we have identified four genes in the PRC2 complex, *Ezh2*, *Suz12*, *Mtf2*, and *Jarid2*, with a role in direct conversion of fibroblasts into iNSCs. In addition to these regulators, we showed that core regulators of pluripotency that contain Pou5f1, Sox2, Nanog, and Sall4 are also involved in the conversion of the fibroblasts into iNSCs. Collectively, these eight factors play an essential role in cell fate decision and pluripotency. Pluripotency was also affected by Tbx3, Klf4, and Foxd3 genes, which regulate *Pou5f1*, *Sox2*, and *Nanog*. In ESCs, it has been discovered that Tbx3, Klf4, and Foxd3 are mainly repressed by PRC2. PRC2 knockdown mice showed an upregulation of these genes and subsequent increase in the expression of core regulatory components of pluripotency including *Pou5f1*, *Sox2*, and *Nanog* [47]. Increases in the expression of Tbx3, Klf4, and Foxd3 inhibit differentiation and guarantee maintenance of pluripotency. Negative regulation of these three genes by PRC2 prepares cells to respond to external stimuli for fate decision and differentiation. However, these genes are absent in our DE gene list. These findings might suggest that they are repressed by PRC2 during this conversion. So, it seems that produced iNSCs efficiently respond to external stimuli for terminal differentiation into neurons, oligodendrocytes, and astrocytes.

## Conclusions

In this study, we have dissected the gene expression regulations during conversion of mouse fibroblasts into iNSCs. Here, using protein-DNA, protein-protein and expression data we have constructed a gene regulatory network. We found 46 TFs were differentially expressed at this conversion based on ChIP enrichment analysis. Applying statistical analysis to the network and evaluating the collaboration of genes in the signaling pathways regulation results in identifications of ten TFs with critical roles in this conversion. *Pou5f1*, *Sox2*, *Nanog* and *Sall4* compose core regulators of pluripotency and *Mtf2*, *Suz12*, *Ezh2* and *Jarid2* form the PRC2 complex. These eight TFs along with *Tcf3* and *Smarca4* are ten TFs that were identified as master regulators. Following analysis of three independent experiments, we have demonstrated *Sox2* and *Ezh2* as the main regulators of direct conversion of mouse fibroblasts into iNSCs. The results of this study may unravel new aspects of direct reprogramming of mouse fibroblasts into iNSCs through introducing new master regulators.

## Additional files

Additional file 1: Table S1. Data sets which were used for comparison of NSCs with astrocytes and neurons. Additional file 2: Table S2. Full list of DE genes in conversion of mouse fibroblasts into iNSCs. Additional file 3: Table S3. Regulatory and protein-protein interactions of whole network. Additional file 4: Table S4. Results of centrality analysis of gene regulatory network using six centrality indexes. Additional file 5: Table S5. List of the most affected signaling pathways and their involved DE genes. Additional file 6: Table S6. Predicting collaboration of TFs in regulation of the most affected signaling pathways.

## Abbreviations

ChEA: ChIP enrichment analysis
ChIP: chromatin immunoprecipitation
DAVID: Databases for Annotation, Visualization and Integrated Discovery
DE: differentially expressed
EB: Embryoid body
ESC: embryonic stem cell
FANMOD: fast network motif detection
iNSCs: induced neural stem cells
iPSC: induced pluripotent stem cell
MEFs: mouse embryonic fibroblasts
NPCs: Neural progenitor cells
PRC2: polycomb repressive complex 2
RMA: robust multi-array averaging
TFs: transcription factors
