# Supplementary material for: Predicting involvement of polycomb repressive complex 2 in direct conversion of mouse fibroblasts into induced neural stem cells
Source: Stem Cell Res Ther. 2015 Mar 21;6(1):42. doi: 10.1186/s13287-015-0045-x (PMC4397673; doi:10.1186/s13287-015-0045-x)
Supplement: Additional file 1: Table S1. — Data sets which were used for comparison of NSCs with astrocytes and neurons. [file 13287_2015_45_MOESM1_ESM.docx]

| Experiment | Comparison | Accession number | Description |
| --- | --- | --- | --- |
| Deng et al., (2013)  [27] | Wild type neural progenitor cells (NPCs) versus neurons | GSE44175 | Microarray data of wild type samples of this study were used. |
| Kim et al. (2009)  [28]  And  Cahoy et al. (2008)  [29] | NSC samples from Kim et al study versus postnatal age day 16 astrocyte samples | GSE12499  GSE9566 | iNSC2, iNSC3, and iNSC5 colons compared with MEF. |
