# Supplementary material for: Predicting involvement of polycomb repressive complex 2 in direct conversion of mouse fibroblasts into induced neural stem cells
Source: Stem Cell Res Ther. 2015 Mar 21;6(1):42. doi: 10.1186/s13287-015-0045-x (PMC4397673; doi:10.1186/s13287-015-0045-x)
Supplement: Additional file 4: Table S4. — Results of centrality analysis of gene regulatory network using six centrality indexes. [file 13287_2015_45_MOESM4_ESM.docx]

| G.Symbol | Degree | Eccentricity | Closenes | Stress | Betweenness | Centroid | Average | Ranke |
| --- | --- | --- | --- | --- | --- | --- | --- | --- |
| Nanog | 2 | 1 | 3 | 1 | 3 | 1 | 1.83 | 1 |
| Pou5f1 | 2 | 1 | 2 | 2 | 6 | 1 | 2.33 | 2 |
| Pparg | 3 | 2 | 4 | 6 | 1 | 1 | 2.83 | 3 |
| Mtf2 | 4 | 3 | 7 | 4 | 2 | 1 | 3.5 | 4 |
| Sox2 | 5 | 1 | 6 | 3 | 7 | 1 | 3.83 | 5 |
| Myc | 7 | 1 | 8 | 5 | 5 | 1 | 4.5 | 6 |
| Suz12 | 1 | 2 | 1 | 11 | 12 | 1 | 4.66 | 7 |
| Tcf3 | 6 | 1 | 5 | 9 | 8 | 1 | 5 | 8 |
| Smarca4 | 8 | 2 | 9 | 7 | 4 | 1 | 5.16 | 9 |
| Ezh2 | 10 | 2 | 10 | 8 | 9 | 1 | 6.66 | 10 |
| Jarid2 | 12 | 3 | 14 | 10 | 10 | 1 | 8.33 | 11 |
| Tet1 | 9 | 2 | 13 | 16 | 15 | 1 | 9.33 | 12 |
| Sall4 | 14 | 1 | 12 | 15 | 21 | 1 | 10.66 | 13 |
| Olig2 | 17 | 2 | 20 | 13 | 13 | 1 | 11 | 14 |
| Trim28 | 11 | 2 | 11 | 22 | 23 | 1 | 11.66 | 15 |
| Wt1 | 16 | 2 | 21 | 17 | 16 | 1 | 12.16 | 16 |
| Mycn | 25 | 2 | 23 | 14 | 14 | 1 | 13.16 | 17 |
| Myb | 28 | 2 | 27 | 12 | 11 | 1 | 13.5 | 18 |
| Mybl2 | 22 | 2 | 22 | 20 | 22 | 1 | 14.83 | 19 |
| Rad21 | 18 | 2 | 15 | 28 | 26 | 1 | 15 | 20 |
| Nr0b1 | 21 | 1 | 16 | 24 | 27 | 1 | 15 | 20 |
| Phc1 | 30 | 2 | 29 | 18 | 18 | 1 | 16.33 | 21 |
| Sin3a | 34 | 2 | 25 | 19 | 20 | 1 | 16.83 | 22 |
| Cebpb | 23 | 3 | 44 | 21 | 19 | 1 | 18.5 | 23 |
| Tfcp2l1 | 20 | 2 | 18 | 36 | 37 | 1 | 19 | 24 |
