# Supplementary material for: Predicting involvement of polycomb repressive complex 2 in direct conversion of mouse fibroblasts into induced neural stem cells
Source: Stem Cell Res Ther. 2015 Mar 21;6(1):42. doi: 10.1186/s13287-015-0045-x (PMC4397673; doi:10.1186/s13287-015-0045-x)
Supplement: Additional file 5: Table S5. — List of the most affected signaling pathways and their involved DE genes. [file 13287_2015_45_MOESM5_ESM.docx]

| Nu | Signaling | N.Genes | *p-value* | genes |
| --- | --- | --- | --- | --- |
| 1 | G-protein coupled receptor signaling pathway | 64 | 4.91E-15 | Abhd6, Adm, Adora2b, Adrb1, Adrb2, Agtr2, Apoe, Arrb2, Bai2, Bai3, Bicd1, Cck, Celsr1, Celsr2, Dtnbp1, Edn1, Ereg, F2r, F2rl1, Fzd1, Fzd2, Gabrb3, Glrb, Gna14, Gnb4, Gng11, Gng13, Gng8, Gngt2, Gpr124, Gpr126, Gpr133, Gpr153, Gpr176, Gpr35, Grk5, Htr3a, Ifi47, Itgb1, Kcnk2, Klk1, Lpar1, Lrp1, Lrrk2, Mrgpre, Npr3, Npy, Pcsk1n, Penk, Plce1, Prokr1, Ptger4, Ptgir, Pth1r, Ramp2, Ramp3, Rgs10, Rgs16, Rgs3, Rgs5, Rgs7, S1pr3, Sphk1, Tac1 |
| 2 | ERK1 and ERK2 cascade | 36 | 1.45E-10 | Arrb2, Bmp4, Bmper, C1qtnf3, Ccl11, Clcf1, Cnksr3, Csf1r, Ctgf, Ctsh, Cyr61, Dusp3, Dusp6, Epha2, Epha7, F2r, F2rl1, Fgf10, Fgf2, Fgf4, Fgfr3, Igf1, Il6, Mbd4, Pdgfra, Pdgfrb, Prkca, Ptger4, Ptpn6, Rgs16, Rps6ka6, Sema7a, St5, Tek, Tlr4, Tnfsf11 |
| 3 | MAPK cascade | 72 | 1.97E-09 | Adrb2, Arrb2, Bmp4, Bmper, C1qtnf3, Cav1, Cbs, Ccl11, Ccl2, Cdh2, Cdon, Clcf1, Cnksr3, Csf1r, Ctgf, Ctsh, Cyr61, Dusp1, Dusp3, Dusp6, Edn1, Epha2, Epha7, F2r, F2rl1, Fgf10, Fgf2, Fgf4, Fgfr3, Flt1, Foxo1, Fzd2, Fzd8, Grem1, Grm4, Igf1, Igfbp4, Il11, Il6, Itgb1, Kit, Lmnb1, Lpar1, Ltbr, Map3k5, Mapk13, Mbd4, Mdfic, Myc, Nenf, Pdcd4, Pdgfra, Pdgfrb, Prkca, Ptger4, Ptpn6, Rbm4, Ret, Rgs16, Rps6ka6, Scg2, Sema7a, Sfrp1, Sox2, St5, Tek, Timp2, Tlr4, Tnfsf11, Wnt16, Wnt5a, Wwc1 |
| 4 | transmembrane receptor protein tyrosine kinase signaling pathway | 66 | 7.62E-09 | Agtr2, Angpt4, Atxn1, Bmp4, Ccl2, Cd59a, Cilp, Csf1r, Ctgf, Dusp6, Efemp1, Enpp1, Epha2, Epha7, Ephb3, Ereg, Errfi1, F3, Fert2, Fgf10, Fgf2, Fgf4, Fgf7, Fgfbp1, Fgfr3, Flt1, Foxc2, Foxo1, Gata3, Gdnf, Gfra1, Ghr, Gpc1, Grb14, Hbegf, Hgf, Hhex, Hhip, Hif1a, Igf1, Igfbp4, Kit, Leprot, Lrp1, Ngef, Ngf, Nrp1, Pdgfc, Pdgfra, Pdgfrb, Plat, Prkca, Prkd1, Ptprf, Ret, Rgs16, Rhoq, Sgpl1, Sirt1, Sulf1, Tdgf1, Tek, Thbs1, Vegfa, Wnt5a, Zfyve28 |
| 5 | BMP signaling pathway | 28 | 3.81E-08 | Acvr2a, Acvr2b, Acvrl1, Bmp2, Bmp4, Bmper, Bmpr1b, Cav1, Cyr61, Fbn1, Foxd1, Fst, Fstl3, Fzd1, Grem1, Hes5, Htra1, Nanog, Nbl1, Notch1, Sfrp1, Smad6, Smad7, Sox11, Sulf1, Tdgf1, Tgfbr3, Wnt5a |
| 6 | transforming growth factor beta receptor signaling pathway | 29 | 1.29E-07 | Acvrl1, Adam9, Arrb2, Bmp2, Bmp8a, Bmpr1b, Ccl2, Cdkn2b, Chst11, Cited1, Col1a2, Col3a1, D0H4S114, Fbn1, Gdnf, Htra1, Itga8, Nodal, Nr3c1, Prdm16, Sirt1, Smad6, Smad7, Sox11, Tdgf1, Tgfb1i1, Tgfb3, Tgfbr3, Thbs1 |
| 7 | Wnt receptor signaling pathway | 52 | 1.37E-06 | Ankrd6, Apcdd1, Bicc1, Bmp2, Cav1, Ccdc88c, Ccnd1, Cdh2, Celsr1, Celsr2, Col1a1, Cthrc1, Dact1, Ddit3, Dkk2, Dkk3, Fgf10, Fgfr3, Frat2, Frzb, Fzd1, Fzd2, Fzd8, Gata3, Grk5, Hhex, Hoxb9, Jup, Lrp1, Mark1, Mdfic, Myc, Nkd2, Notch1, Nrarp, Peg12, Pitx2, Ppap2b, Rspo3, Sall1, Sfrp1, Snai2, Sox2, Sulf1, Tdgf1, Tgfb1i1, Tle2, Tle4, Vax2, Wisp1, Wnt16, Wnt5a |
| 8 | cytokine-mediated signaling pathway | 29 | 1.92E-05 | Cav1, Ccl2, Csf1r, Ereg, F2rl1, Fert2, Gas6, Iigp1, Il1r1, Il6, Il6st, Irak2, Jak1, Kit, Klf6, Krt8, Mbd4, Pparg, Ptpn6, Ptprf, Ptprn, Rps6ka5, Sigirr, Slit2, Slit3, Sphk1, Stat4, Tnfsf11, Wnt5a |
| 9 | Rho protein signal transduction | 30 | 3.75E-04 | Adrb1, Apoe, Arhgap6, Arhgef3, Ccl11, Cdh2, Celsr1, Col1a2, Ctnnal1, Dlc1, E130306D19Rik, Ect2, Epha1, Epha2, Ephb3, F2r, F2rl1, Farp1, Fgd5, Fzd2, Lpar1, Mcf2l, Ngef, Pkp4, Prex1, Rhoj, Scrib, Spata13, Syde1, Thy1 |
| 10 | Ras protein signal transduction | 39 | 0.02 | Adap1, Adrb1, Apoe, Arhgap6, Arhgef3, Asap3, Ccl11, Ccna2, Cdh2, Celsr1, Col1a2, Csf1, Ctnnal1, Dlc1, E130306D19Rik, Ect2, Epha1, Epha2, Ephb3, F2r, F2rl1, Farp1, Fgd5, Fgf10, Fzd2, Igf1, Lpar1, Mcf2l, Ngef, Pkp4, Plce1, Prex1, Rasa3, Rhoj, Rras2, Scrib, Spata13, Syde1, Thy1 |
